# Differentiation Capacity of Mouse Dental Pulp Stem Cells into Osteoblasts and Osteoclasts

**Published:** 2014-02-03

**Authors:** Shabnam Kermani, Rohaya Megat Abdul Wahab, Intan Zarina Zainol Abidin, Zaidah Zainal Ariffin, Sahidan Senafi, Shahrul Hisham Zainal Ariffin

**Affiliations:** 1 School of Biosciences and Biotechnology, Faculty of Science and Technology, University Kebangsaan Malaysia, 43600 Bangi, Selangor, Malaysia; 2 Department of Orthodontics, Faculty of Dentistry, University Kebangsaan Malaysia, Jalan Raja Muda Abdul Aziz, 50300 Kuala Lumpur, Malaysia; 3 School of Biology, Faculty of Applied Sciences, University Teknologi MARA, 40450 Shah Alam, Selangor, Malaysia

**Keywords:** Dental Pulp, Stem Cells, Differentiation, Osteoblasts, Osteoclasts

## Abstract

**Objective:**

Our research attempted to show that mouse dental pulp stem cells (DPSCs) with characters such as accessibility, propagation and higher proliferation rate can provide an improved approach for generate bone tissues. With the aim of finding and comparing the differentiation ability of mesenchymal stem cells derived from DPSCs into osteoblast and osteoclast cells; morphological, molecular and biochemical analyses were conducted.

**Materials and Methods:**

In this experimental study, osteoblast and osteoclast differentiation was induced by specific differentiation medium. In order to induce osteoblast differentiation, 50 μg mL-1 ascorbic acid and 10 mM β-glycerophosphate as growth factors were added to the complete medium consisting alpha-modified Eagle’s medium (α-MEM), 15% fetal bovine serum (FBS) and penicillin/streptomycin, while in order to induce the osteoclast differentiation, 10 ng/mL receptor activator of nuclear factor kappa-B ligand (RANKL) and 5 ng/mL macrophage-colony stimulating factor (M-CSF) were added to complete medium. Statistical comparison between the osteoblast and osteoclast differentiated groups and control were carried out using t test.

**Results:**

Proliferation activity of cells was estimated by 3-[4,5-dimethylthiazol-2-yl]-2,5 diphenyl tetrazolium bromide (MTT) assay. Statistical results demonstrated significant difference (p<0.05) between the control and osteoblastic induction group, whereas osteoclast cells maintained its proliferation rate (p>0.05). Morphological characterization of osteoblast and osteoclast was evaluated using von Kossa staining and May-Grunwald- Giemsa technique, respectively. Reverse transcription-polymerase chain reaction (RTPCR) molecular analysis demonstrated that mouse DPSCs expressed *Cd146* and *Cd166* markers, but did not express *Cd31*, indicating that these cells belong to mesenchymal stem cells. Osteoblast cells with positive osteopontin (Opn) marker were found after 21 days, whereas this marker was negative for DPSCs. *CatK*, as an osteoclast marker, was negative in both osteoclast differentiation medium and control group. Biochemical analyses in osteoblast differentiated groups showed alkaline phosphatase (ALP) activity significantly increased on day 21 as compared to control (p<0.05). In osteoclast differentiated groups, tartrate-resistant acid phosphatase (TRAP) activity representing osteoclast biomarker didn’t show statistically significant as compared to control (p>0.05).

**Conclusion:**

DPSCs have the ability to differentiate into osteoblast, but not into osteoclast cells.

## Introduction

Current research trends of making use of postnatal stem cells as a source of stem cell to increase the quality and quantity of functional cells have been very helpful in the field of regenerative medicine (1). Before treatment, speed, accurate, impressionable material and suitable growth factors are important considerations that can be used during in vitro stem cells culture to ensure successful utilization for medical purposes. Among adult stem cells, it seems stem cells that are found in teeth may be an ideal source for regenerative medicine because through clinical practice like tooth extraction, we can have an available source of stem cells. Little is known about the characteristics of stem cells, especially dental stem cells; however, few studies were done to use these cells for dental tissue regeneration (2). Teeth can be a unique and accessible source of stem cell in applied researches and tissue engineering with less ethical consideration (3). Therefore, locating a viable source of stem cells and preparing the condition for differentiation are fundamental in stem cell research.

Bones and cartilages form skeletal system in the body. These tissues are known as connective tissues. Bone matrix has the unique ability of calcification. Two types of bone cells known as osteoblasts and osteoclasts play important roles in bone-forming and bone-resorbing, respectively (4). Osteoblasts are derived from osteoprogenitor cells that originate at the bone marrow. These osteoprogenitors are immature progenitor cells that participate in bone formation. Bone removal and renewal are needed to maintain the homeostasis in bone tissues. Therefore, a combination of osteoblasts and osteoclasts activities can improve this process (5). Since osteoblasts are derived from mesenchymal stem cells and dental pulp stem cells are a type of mesenchymal stem cells, their ability to differentiate to osteoblasts and osteoclasts was investigated. In this study, to identify osteogenic capacity of dental pulp stem cells and to characterize their differentiated cells, dental pulp stem cells were cultured in osteoblast and osteoclast medium for 21 days.

## Materials and Methods

This experimental study was conducted on healthy mice aged 6-8 weeks. Healthy female mice were obtained from the Animal House of universiti kebangsaan Malaysia (UKM) approved by The Faculty of Science and Technology University Kebangsaan Malaysia Research Ethics Committee (FST-UKM REC). The mice were killed with inhalation chloroform, and the tooth extraction was performed by using dental pliers. Teeth were immediately placed in 1X phosphate buffer saline (PBS, Sigma, USA) containing 1% (v/v) penicillin streptomycin (Invitrogen, USA) and kept at 4˚C. The teeth were, thereafter, transferred to cell culture lab for extraction of the pulp tissues.

Dental pulp extraction was carried out using surgical forceps and scalpel. The apical part of the teeth was removed with a scalpel, and the dental pulp was removed with a forceps. The dental pulp tissue was placed in 4 unit of collagenase type 1 (Sigma-Aldrich, Inc., USA) at 37˚C. After an hour of enzyme reaction, alpha-modified Eagle’s medium (α-MEM, Invitrogen, USA) was pipetted into the cell mixture, thus a single cell form was obtained. Then, α-MEM containing 20% (v/v) fetal bovine serum (FBS; Biowest, South America) was added to the cultivation medium, and it was centrifuged at 1200 g for 10 minutes. The obtained cell plate was once again mixed with the aforementioned cultivation medium, and then transferred to a T25 flask containing α-MEM supplemented with 20% (v/v) FBS. The flasks were, thereafter, incubated at a temperature of 37˚C, humidity of 95% and 5% CO2. After 24 hours of incubation, suspended cells were removed from the medium as the dish was washed with 1X PBS solution.

### Osteoblast cells differentiation

Differentiation of dental pulp stem cells was carried out with the addition of differentiating factors to the complete growth medium during cell plating in 24-well plates. Approximately, 1×10^5^ cells were prepared in each of the wells for the osteoblast differentiation analysis. Thereafter, 50 μg/mL ascorbic acid (Sigma, USA) and 10 mM β-glycerol phosphate (w/v, Sigma, USA) were added to induce differentiation of dental pulp stem cells into osteoblast cells. Culture medium with no added differentiation induction factor was taken as negative control.

### Osteoclast cells differentiation

Approximately, 1×10^5^ cells were prepared in each of the 24-wells for the osteoclast differentiation analysis. For osteoclast differentiation, 10 ng/mL RANKL (Peprotech, USA) and 5 ng/mL macrophagecolony stimulating factor (M-CSF, Peprotech, USA) were added to the complete medium to induce differentiation of dental pulp stem cells into osteoclast cells. Culture medium with no differentiation induction factor was taken as negative control

### RNA extraction

In this method, Trizol was used to extract total RNA. After centrifugation, cell pellet was resuspended in 1 mL of 100% (v/v) Trizol (Sigma, USA) for 5 minutes at room temperature to lyse the cells. Then, 200 μL of chloroform was added, and the sample was incubated for a 10-12-minute period at room temperature after being vortexed for 15 seconds. The sample was then centrifuged at 12000 g for 15 minutes at 4˚C to complete the extraction process, resulting in three phases including protein (organic phase), DNA (middle phase), and RNA (colorless phase). RNA was transferred to diethyl pyrocarbonate (DEPC)-treated tubes, and 0.5 mL of 100% (v/v) isopropanol was added to precipitate the RNA. The mixture was left undisturbed for 10-12 minutes at room temperature, and then centrifuged at 12000 g at 4˚C for 10 minutes. After the supernatant was discarded, 1 mL of 75% (v/v) ethanol was added to the pellet, and the mixture was centrifuged at 7500 g at 4˚C for 5 minutes. After the supernatant was discarded for the second time, the washed RNA pellet was left to dry at room temperature for 5-10 minutes. The RNA pellet was dissolved with 25 μL of nuclease-free-water and incubated at 55˚C for 5-10 minutes. Then, RNA sample was stored at 80˚C until used.

### Reverse transcription-polymerase chain reaction for dental pulp stem cells, osteoblast and osteoclast

Following RNA measurement using biophotometer machine, 1 μg of RNA template was used for reverse transcription-polymerase chain reaction (RT-PCR) procedure. The RT-PCR (kit Access Quick^TM^ RTPCR system, Promega, USA) reaction mix consists of 5X reaction buffer AMV/Tfi, dNTP mix (10 mM), forward and reverse primers (50 pmol), avian myeloblastosis virus (AMV) reverse transcriptase (5 U/μL), DNA polymerase Tfi (5 U/μL) and MgSO_4_ (25 mM). Eventually, RT-PCR reaction was conducted using mouse dental pulp RNA with each reaction along with a specific pair of primer for *Cd166*, *Cd146* and *Cd31*. For dental pulp stem cells differentiated into osteoblast and osteoclast cells, RT-PCR reaction was conducted using osteopontin (OPN) as osteoblast cell marker and cathepsin as osteoclast cell marker. Primer sequences used in this procedure are listed in table 1

Reverse transcription was carried out at 45˚C for 45 minutes, and then AMV reverse transcriptase (5 U/μL) was deactivated at 94˚C for 2 minutes for the initiation of primary cDNA synthesis. Forty cycles of denaturation at 94˚C for 30 seconds, one minute at optimal annealing temperature depending on primer design (Table 1) and two minutes of primary extension at 68˚C was conducted for secondary cDNA synthesis and PCR amplification. Final extensions at 68˚C for seven minutes were performed. The amplified products were separated using 1% (w/v) agarose gels (Promega, USA). The gels were stained with ethidium bromide and examined with UV-analyzed using Alpha Imaging System (Alpha Innotech, USA).

**Table 1:** Primer sequence and the annealing temperature of RT-PCR.


Name	Forward primer	Reverse primer	Accession No.	Length (bp)	Ann. T (°C)

**Cd 146**	^5`^GGACCTTGAGTTTGAGTGG ^3`^	^5`^CAGTGGTTTGGCTGGAGT ^3`^	NM_023061	479	60.0
** Cd 166**	^5`^AACATGGCGGCTTCAACG ^3`^	^5`^GACGACACCAGCAACGAG ^3`^	NM_009655	630	61.0
** Gapdh**	^5`^CAACGGCACAGTCAAGG ^3`^	^5`^AAGGTGGAAGAGTGGGAG ^3`^	NM_008084	717	62.0
** Cd 31**	^5`^GGTCTT GTCGCAGTATCAG ^3`^	^5`^ATGGCAATTATCCGCTCT ^3`^	NM_001032378.1	355	58.0
** Opn**	^5`^ CACTCCAATCGTCCCTACA^3`^	^5`^ GCTGCCCTTTCCGTTGTT ^3`^	NM_009263.2	234	62.0
** CatK**	^5`^GGCAGGGTCCCAGACTCCAT^3`^	^5`^GTGTTGGTGGTGGGCTAC ^3`^	NM_007802.3	350	53.0


### Cell staining for osteoblast cells

Von Kossa staining, used to identify osteoblast cells, was conducted on days 1, 14 and 21. Approximately, 10% (v/v) formalin (R&M Chemicals, UK) in phosphate buffer saline (PBS, Sigma, USA) was added into each well and incubated for 30 minutes. Then, cultured cells were washed with deionized water 3 times, followed by cells incubation with 100% (v/v) silver nitrate solution (Sigma, USA) for 30 minutes in dark room. After this cells were washed thrice with deionized water, followed by the addition of 10% (w/v) sodium carbonate (Fisher Scientific, UK) solution in 25% (v/v) formalin, it was incubated for 5 minutes at room temperature. Thereafter, the cultured cells were washed with deionized water three times before the addition of 5% (w/v) sodium thiosulphate (Sigma, USA) solution. After 2 minutes, cultured cells were washed again with deionized water three times. Cells which were able to differentiate to mature osteoblast cells were stained by von Kossa staining. Characteristically in this technique, calcium materials are identifiable by black or brown color deposits, so osteoblast cells were visualized as brown or dark brown color.

### Osteoclast staining using May-Grunwald-Giemsa

Approximately, 1×10^5^ cells were seeded in 24- well plates, induced into osteoclast cells for 21 days and washed with 1X PBS. May-Grunwald-Giemsa (MGG) staining was carried out with May-Grunwald’s eosin methylene blue (Merck, Germany) and Giemsa solution (Sigma, Germany). Osteoclast staining was carried out with 100% (v/v) May-Grunwald for 4 minutes. Approximately, 4% (v/v) Giemsa (Sigma, Germany) was added to cells in each of the 24-well plates after being induced with osteoclast medium for 4 minutes and rinsed thrice with distilled water. Excess dye was wiped off the plates and air-dried. This was followed by microscopic examination of cells.

### Biochemical assay (alkaline phosphatase assay and telomeric repeat amplification protocol assay)

#### cell preparation for biochemical assay

The enzyme activity of alkaline phosphatase (ALP) was assayed in this experiment. Approximately, 1×10^3^ cells/well of DPSCs were seeded in 96-well plates. This was done by treating cells with 0.25% (v/v) trypsin/ethylenediaminetetraacetic acid (EDTA) for 5 minute, followed by centrifugation at 1200 g for 10 minutes. After 1, 5, 7, 10, 14 and 21 days of culture with differentiation medium (osteoblast specific differentiation medium) and control medium (complete medium), the ALP activity of DPSCs was determined via ALP assay using alogots, taken every two days. The control medium and differentiate medium were removed every two days for ALP assay. Cell preparation for telomeric repeat amplification protocol (TRAP) assay is similar to that of ALP assay.

#### i. Alkaline phosphatase activity for osteoblast cells

For evaluation of alkaline phosphatase activity, the cells were washed with PBS, and then 0.1 M NaNO3- Na2Co3 buffer (pH 10.0, w/v, R&M, UK) containing 1% (v/v) Triton X100 (Sigma, USA) and 2 mM MgSO_4_ (w/v, Sigma, USA) was added to the cells. Thereafter, 6 mM p-nitrophenylphosphate (w/v, Sigma, USA) was added as substrate to each 96-well plates, and the cells were incubated for 30 minutes at 37˚C. To stop the enzyme substrate reaction, 1.5 M NaOH (Sodium hydroxide, w/v, Sigma, USA) was added to each 96-well plates. Optical density measurement was then taken at a wavelength of 405 nm with a spectrophotometer (Cary Varian, Australia).

#### ii.Tartrate-resistant acid phosphatase assay for osteoclast cells

The cells were washed with PBS and incubated with 50 μL sodium acetate buffer (50 mM, pH=5.5) containing Triton X-100 (0.1% v/v) at -20˚C for 40 minutes. TRAP enzyme activity was assayed using p-nitrophenyl phosphate (pNPP) as substrate in an incubation medium containing 10 mM pNPP, 0.1 M sodium acetate (pH=5.8), 0.15 M KCl, 0.1% (v/v) Triton X-100, 10 mM sodium tartrate, 1 mM ascorbic acid and 0.1 mM FeCl3. The p-nitrophenol was liberated into p-nitrophenylate after 1 hour of incubation at 37˚C, and the reaction was stopped by adding 500 μL of 0.6 M NaOH. The absorbance was immediately taken at 405 nm using a spectrophotometer (Cary Varian, Australia).

### Statistical analysis

Statistical comparison between the differentiated groups (osteoblast and osteoclast differentiation medium) and control groups were carried out by using paired t test. Each experiment was repeated independently for three times where the cells were isolated from three different groups of mice. Also, p values less than 0.05 were considered as statistically significant.

## Results

### Morphological analysis

**Fig 1:**
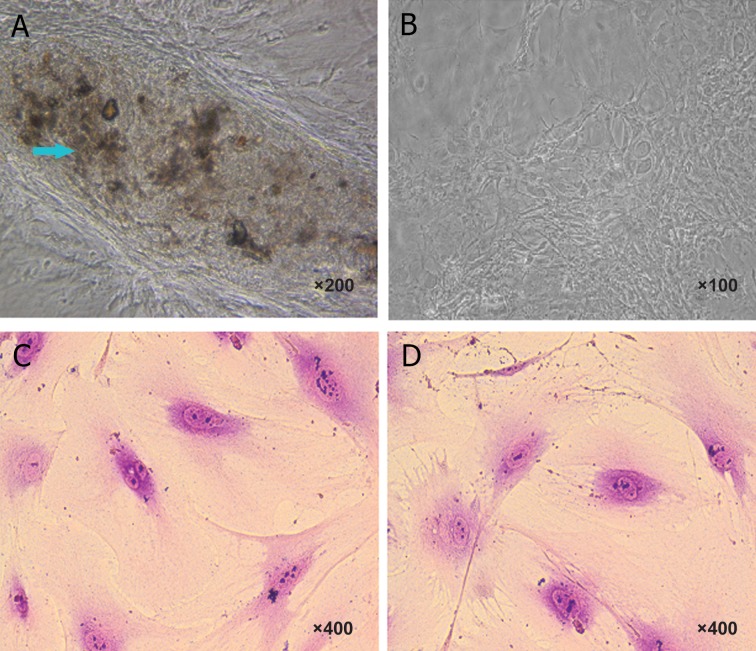
Characteristics of osteoblast and osteoclast derived from mouse dental pulp stem cells. Morphology of stem cells after 21 days exposed to osteoblast induction medium (A). The morphology of mouse dental pulp cell before induction (B). The blue arrows show calcium salt with a dark brown color, whereas the control group that was not exposed to osteoblast induction did not show any calcium nodule. In osteoclast induction medium, the morphology of mouse dental pulp cell after induction (C). Morphology of stem cells before exposing to osteoclast induction (D). Osteoclast cells were stained by May-Grunwald-Giemsa. The staining indicated multinucleated cells were found in dental pulp stem cells cultured in both medium, i.e., osteoclast differentiation medium and complete medium (control).

Morphological characterizations of osteoblasts were evaluated by von Kossa staining. After staining with von Kossa, the production of mineralized matrix nodules with dark colors indicating osteoblast differentiation of mesenchymal stem cells was observed. This technique was successful because it was used to detect calcium material. In this staining, calcium mineralization capacity was identified by dark brown deposits. In fact, osteoblast cells were revealed by von Kossa silver staining and visualized as metallic silver. Dental mesenchymal stem cells were negative for von Kossa staining because there were no calcium deposits in their culture. To differentiate dental stem cells to osteoblast, the fourth cell passage was used. Osteoblasts derived from mouse dental pulp stem cells of a 21-day culture exposed to osteogenic induction revealed the presence of produced calcium nodules detected as dark colour deposits following von Kossa staining (Fig 1A). On the other hand, in control group without exposure to osteogenic induction, there was no calcium nodule formation or detection (Fig 1B).

Morphological characterizations of osteoclast cells were evaluated by May-Grunwald-Giemsa technique. In this staining, the nucleus was stained by May- Grunwald stain, while the cytoplasm was stained with Giemsa stain. Osteoclast cells morphologically appeared as mature multinucleated cells. Our studies showed that multinucleated cells were found in dental pulp stem cells cultured both in osteoclast differentiated medium (in the presence of M-CSF and RANKL) and in the control group (cultured in complete medium only). Our observation showed that dental pulp stem cells have multinucleated cells even before stimulation into osteoclast cells (Fig 1 C, D). Therefore, we could not prove that the existence of multinucleated cells was related to osteoclast differentiation. Furthermore, after using M-CSF and RANKL as osteoclast inducers, there were no phenotype changes during differentiation. To confirm this, biochemical assays of TRAP activity and molecular analysis of *CatK* activation were employed.

### Molecular analysis

**Fig 2:**
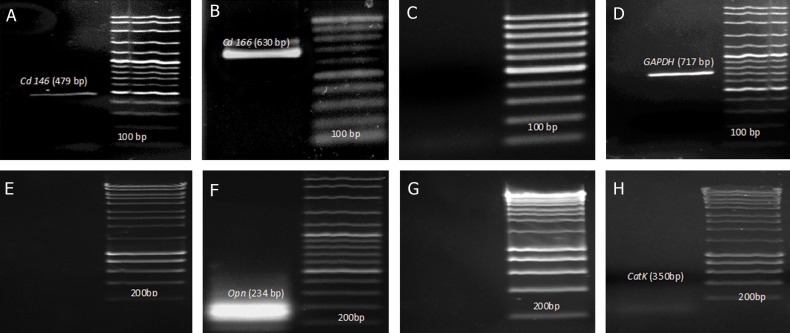
RT-PCR analysis of mouse dental pulp stem cells. RT-PCR molecular analysis indicated that mouse dental pulp stem cells (DPSCs) expressed (A) *Cd146* (479bp), (B) *Cd166* (630 bp) and not (C) d31 (355 bp). Absence of hematopoietic stem cell markers indicate that these cells are of mesenchymal origin. Expression of (D) Gapdh (glyceraldehyde 3-phosphate dehydrogenase) as a house keeping gene (717bp). For RT-PCR analysis of osteogenic capacity of mouse dental pulp stem cells, two differentiation medium were used, i.e., osteoblast and Osteoclast medium. Osteoblast differentiation medium was used for differentiate into osteoblast cells. Opn (234bp) as an osteoblast marker, which absent before osteoblast induction (E), however was found present after 21 days exposure to osteoblast differentiation (F). *CatK* (350 bp) as an osteoclast marker was not detected before induction (G) and after exposure to osteoclast differentiation (H).

RT-PCR analysis results indicated that mouse dental pulp cells expressed *Cd146* and *Cd166* as mesenchymal stem cells markers (Fig 2A, B), whereas they did not express *Cd31* as a hematopoietic stem cell markers (Fig 1C). In this study, the capability of dental pulp stem cells for expression of the osteoblastic markers when the cells were cultured with osteoblast differentiation medium was investigated by RT-PCR. The cells induced with osteoblast differentiation medium were found to be positive for Opn marker after 21 days, whereas this marker was negative for dental pulp stem cells (Fig 2E, F). However, RT-PCR analysis on isolated RNA from dental pulp stem cells after 21 days in osteoclast differentiation medium showed that *CatK* marker was not detected after osteoclast differentiation. This was similar to the control group (Fig 2G, H).

### MTT analysis

**Fig 3:**
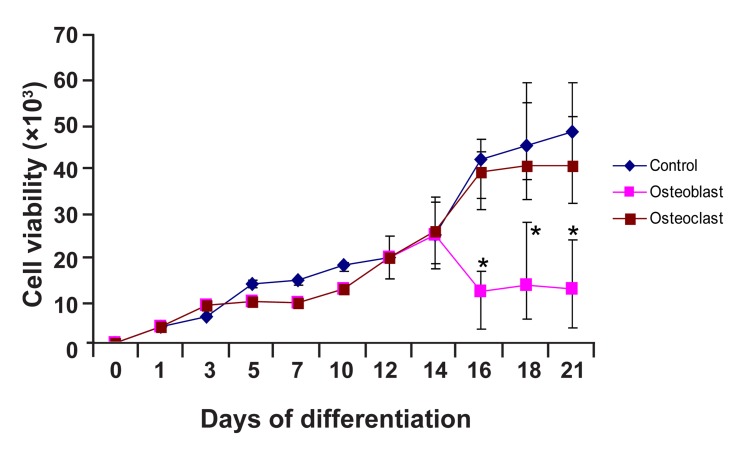
MTT osteogenic differentiation analysis. Cell viability with MTT assay during differentiation stage showed that the proliferation ability of osteoblast differentiated cell is weaker as compared to the control. The results were presented as mean ± SD. Statistical analysis was conducted by t test. Statistical results demonstrated significant difference (*) between the control and osteogenic induction group between day 16 and day 21 (n=3).

The proliferative activity of the cell cultures in differentiation medium was estimated by 3-(4, 5-dimethylthiazol-2-yl)-2, 5 diphenyl tetrazolium bromide (MTT) assay. Cell viability assay with MTT showed that differentiated cells treated with osteogenic medium maintained their growth rate in comparison with control group (culture in AMEM with 15% v/v FBS). However, after 16 days, growth rate of cells cultured in osteoblast differentiation medium decreased, but growth still continued. This showed that these cells were alive during differentiation into osteoblast. Differentiated cells were weaker in their proliferation ability as compared with the control group (including AMEM with 15% v/v FBS) due to differentiation conditions. Statistical analysis revealed significant difference (p<0.05) between the control and osteoblastic induction group. However, cells cultured in osteoclast differentiation medium maintained their proliferation at a rate similar with cells cultured in complete medium (i.e. control group). Statistical analysis also showed that there were no significant difference (p>0.05) in this rate among cells cultured in osteoclast differentiation medium as compared to the control group (Fig 3).

### Alkaline phosphatase analysis

**Fig 4:**
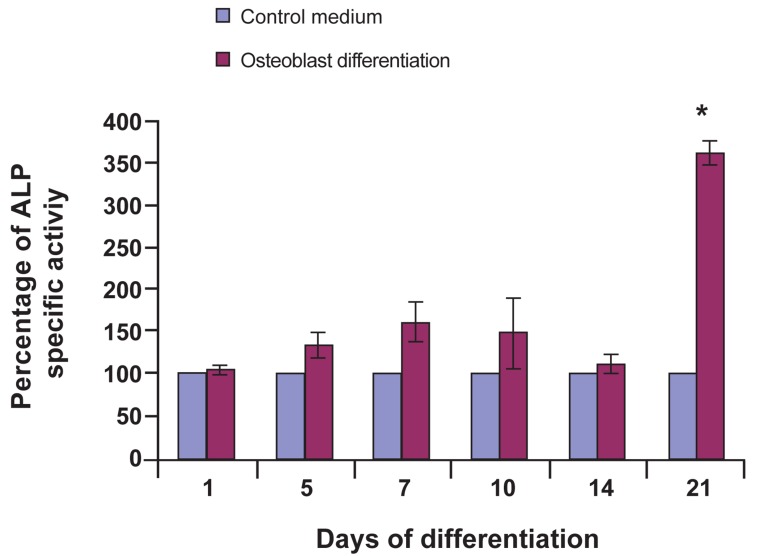
Alkaline Phosphatase (ALP) profile of DPSC in osteoblast differentiation medium. Statistical analysis using paired t test. Comparison of data between dental pulp stem cells as a controls group and dental pulp stem cells induced to osteoblast cell as a differentiated groups showed significant difference (*) only on day 21 (p<0.05, n=3).

Changes in alkaline phosphatase (ALP) enzyme activity during osteoblast differentiation were examined using ALP assay. Results showed that, after culturing dental pulp stem cells in osteogenic induced medium for 21 days, most of the cells became alkaline phosphatase positive as compared to control group cells treated with AMEM and 15% (v/v) FBS. Alkaline phosphatase activity on day 21 increased drastically as compared to that on day 14. Statistic analysis showed significant increase in alkaline phosphatase activity as compared to control group (p<0.05) within the same period (Fig 4). Alkaline phosphatase activities clearly support previous observations (recorded during von Kossa staining) that mineralized nodules appeared on day 21 (Fig 1A).

### Tartrate-rResistant aAcid pPhosphatase analysis

**Fig 5:**
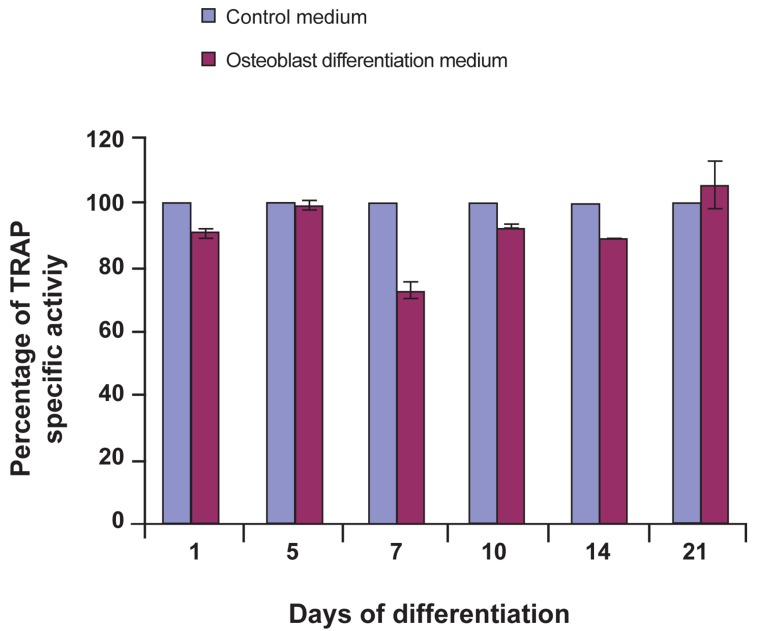
TRAP profile of DPSC in osteoclast differentiation medium. Statistical analysis using paired t test. Comparison of data between dental pulp stem cells as a controls group and dental pulp stem cells induced to osteoclast cell as a differentiated groups didn’t show significant difference throughout 21 days of analysis (p>0.05, n=3).

Tartrate-resistant acid phosphatase (TRAP) assay was used for osteoclast differentiation. When the dental pulp stem cells were cultured in osteoclast differentiation medium, TRAP activities remained constant as was observed among the control cells (Fig 5). TRAP activity, represented osteoclast biomarker, show no statistically significant (p>0.05) changes during culture in osteoclast medium. This indicated that DPSC didn’t differentiate to osteoclast in the present of RANKL and MCSF, osteoclast induction factors, as compared with control group, i.e. cell cultured in control medium at similar period.

## Discussion

MSCs are useful for producing specific cell under specific culture conditions and they offer many advantages. For instance, bone marrows MSCs have more potential to generate osteoblast cells as compared with other MSCs. The other advantages of using MSCs include their ease of isolation from bone marrow, transplantation without rejection and their *in vitro* expansion potentiality. In tissue engineering, stimulation and increasing passage number might help to prevent premature cell aging and cell inactivity (6, 7). Some studies have been conducted on stem cell differentiation to bone cells. For instance, Gronthos et al. (8) differentiated human adult dental pulp stem cells to adipocytes and osteoblasts. In addition, Nakashima et al. (9) differentiated similar cells to odontoblasts and osteoblasts. On the other hand, Miura et al. (3) differentiated human deciduous dental pulp to adipocytes and osteoblasts. Our study proved that mouse dental pulp stem cells as a type of mesenchymal stem cells have the ability to differentiate into osteoblast cells.

The mammalian tissue culture system has to follow physiological environment and mimic the in vivo situation to produce the best cell culture medium conditions for cell growth and cell activity. Therefore, *in vitro* culture systems can be supplemented with various proteins and factors to optimize cell proliferation and cell differentiation (10, 11). Reagents like ascorbic acid 2 phosphate together with β-glycerolphosphate have been used in osteoblast differentiation of bone marrow mesenchymal stem cells and human dental pulp. After 10 days induction by these factors, calcium nodules were generated and molecular investigations showed the differentiation of these cells to osteoblast cells (2, 12).

Ascorbic acid as antioxidants is needed to retain and expand MSC differentiation ability. The other benefit of using this chemical is to reserve proliferation ability without the loss of phenotypes. To find the best concentration of ascorbic acid to be used for this purpose, MSCs were cultured in various concentrations (13, 14). Choi et al. (14) showed that 5-250 μM concentrations of ascorbic acid will not decrease cell density or cause morphological changes.

Hence, they remain non-toxic in this range of concentration. The right concentration of ascorbic acid plays a key role in osteoblast culture systems during differentiation and it is necessary for the expression of osteoblastic markers and for mineralization. In our study, 283 μM (50 μg/mL) ascorbic acid was used and this concentration promoted cell proliferation during differentiation without any associated toxic effect. Takamizawa et al. (15) indicated that ascorbic acid can affect the proliferation and differentiation of human osteoblast-like cells. Their result showed that both ascorbic acid and ascorbic acid 2-phosphate have the ability to stimulate the cells in the expression of osteoblast differentiation markers and the ability to increase alkaline phosphatase activity during differentiation. Takamizawa et al. (15) also showed that ascorbic acid influences the differentiation of MSC and can improve the ability of these cells to proliferate without causing a loss in the differentiation capacity. In our study, when dental pulp stem cells were cultured in osteogenic medium containing ascorbic acid, osteopontin (OPN), as an osteoblast marker, was expressed during osteoblast differentiation besides an increase in alkaline phosphatase activity.

β-glycerophosphate as phosphate donor has a key role in osteoblast differentiation. Osteoblastic cells formation needs an activation to start mineralization in their matrix, hence β-glycerophosphate induces this type of activation. Laurencin et al. (16) indicated that if the media is not supplemented with β-glycerophosphate, mineralization will not occur. When the cells were cultured in osteogenic media in the presence of β-glycerophosphate, mineralized matrix was formed after 21 days. Our study conforms with the observation of Laurencin et al. (16), as mineralized matrix was formed after 21 days when β-glycerophosphate was used in osteoblast differentiation medium.

The differentiated osteoblasts cells were observed to activate OPN and Alp genes. Although bone marrow and dental pulp cells are similar in that they belong to mesenchymal stem cells group, there are differences in the number of colonies formed in the culture (17). Guo et al. (18) evaluated ALP enzyme activity and calcium content during osteogenic differentiation. ALP values and calcium content of the osteogenic cells slightly increased as compared to the first day. Another study by Wu et al. (19) showed that ALP activity in the extracellular matrix increased when the osteoblast cells were matured. In addition, Huang et al. (20) indicated that increase in OPN is concomitant with alkaline phosphatase activity and mineral deposition, which agrees with our result. In our study, in addition to calcium deposition occurring during this period, alkaline phosphatase activity was at an observably high level when the OPN was expressed.

Alkaline phosphate is one of the proteins expressed during *in vitro* differentiation phase occurring between 11 to 25 days (21, 22). In our study, ALP activity was significantly increased as compared to control groups after 21 days following the addition of osteogenic medium. However, Zhao et al. (23) and Yazid et al. (24) showed that during the differentiation of MC3T3-E1 and mouse suspension mononucleated cells into osteoblast, ALP enzyme activities were highest at day 14 when the cells were cultured in differentiation media. These data are in accordance with the findings of Nourbakhsh et al. (25), who reported that ALP activity increased after 3 weeks of osteoblast differentiation in dental pulp of exfoliated human deciduous teeth. Investigation of alkaline phosphatase activity in our study also prove that, there was a statistically significant difference (p<0.05) between alkaline phosphatase activities in cells under treatment of dental pulp stem cells during differentiation into osteoblast cells, as compared to the control group on the 21st day.

One of the osteoblasts differentiation specifications is calcium formation. In our study, calcium nodules were observed with dark brown color through von Kossa staining. The role of temporary proliferation in bone formation is very crucial since high proliferation at this stage results in the increase of bone mass (22). In our study, calcium nodules were formed after 21 days. It can be concluded that mouse dental pulp stem cells have the potency to differentiate to osteoblasts or bone cells. The probable explanation for this claim is that the cells tend to preserve their proliferation ability and viability during differentiation. However, after the 16th day, the ratio of viability among differentiated cells as compare to control group or undifferentiated cells was significantly diminished as indicated in figure 3.

OPN as the major non-collagenous bone proteins, plays a role in bone remodeling and bone forming (26). It is secreted as a calcium-binding glycophosphoprotein that are found in cells such as osteoblasts, endothelial, smooth muscle cells, lymphocytes and macrophages (27). In addition, OPN has been shown to regulate the differentiation of rat bone marrow-derived into osteoblastic cells (26). In our study, expression of OPN in dental pulp stem cells proved successful differentiation of DPSC into osteoblast cells. Molecular investigation in this study showed that osteopontin marker was expressed 21 days after bone induction. With regard to osteopontin bone marker before and after differentiation (Fig 2E, F), it can be seen that this marker was expressed 21 day after nodules were formed (Fig 1A). Investigation of alkaline phosphatase activity is a proof. In this direction, there was a statistical significant difference (p<0.05) between alkaline phosphatase activities in cells under treatment of bone induction, and the control group on the 21st day (Fig 4). This study, therefore, demonstrates that DPSCs are a kind of stem cells with osteogenic differentiation ability. Cell viability during the differentiation into osteoblast also showed that these cells preserved their viability during differentiation. However, after 16 days, the ratio of viability among differentiated cells as compared to the control cells was significantly decreased (p<0.05) indicating that these cells lose their ability to proliferate during the differentiation process (Fig 3).

Our study indicated that dental pulp stem cell could not be differentiated into osteoclast cells. Our observation showed that dental pulp stem cells have multinucleated cells even before stimulation into osteoclast cells (Fig 1C and D). Therefore, we could not prove that the existence of multinucleated cells is related to osteoclast differentiation. Furthermore, after using M-CSF and RANKL as osteoclast inducers, there was no phenotype change during differentiation. To confirm this result, biochemical assays and molecular analysis of *CatK* were employed. TRAP is the marker enzyme that is utilized to recognize osteoclast cells activity. It regulates cell function as an extracellular matrix protein. High expression of *Catepsin K* and trap in rabbit osteoclast have been reported in a previous study (28). Vaananen et al. (29) indicated the resorption process in lacuna by a recognizable high expression of *CatK* in osteoclast cells. Expression of *CatK* happens during the resorption of calcium deposits in calcified tendonitis (30). In our study, no activation of *CatK* was observed. This indicated that DPSC were unable to differentiate into osteoclast *in vitro*.

Studies by Yazid et al. (24) showed that TRAP was secreted during differentiation of peripheral blood mononucleated cells into osteoclast cells after 10 days. However, in our study, during osteoclast differentiation, TRAP activity did not significantly increase. Although Yazid et al. (24) claimed that MC3T3-E1 is an osteoblast progenitor committed to a specific lineage, such claim could not be made in this study because DPSCs were not able to differentiate into osteoclasts. It means that DPSC lacks of proper osteoclast *in vitro* differentiation factor, or do not posses osteoclast differentiation potentiality, and thus do not have the ability to generate osteoclasts (Fig 1C, D). In terms of the characteristics of the osteoclasts, larger sized multinucleated cells were observed in both control groups and osteoclast differentiated groups in DPSCs which normally have multinucleated cells without any stimulus. In molecular investigation conducted during our study, *CatK* marker was also not activated when DPSC was cultured in osteoclast differentiation medium.

In summary, genetic marker studies on osteoblast and osteoclast differentiated cell type have shown that the activation of Opn is an indication that the cells have differentiated into osteoblasts, while the non activation of *CatK* indicates these cells have not differentiated into osteoclast. According to ALP and TRAP assay, ALP result showed that, after 21 days of culturing dental pulp stem cells with osteoblastic induced medium, most of the cells became alkaline phosphatase positive as compared to the control group. In contrast, for TRAP assay, comparison of data between controls and osteoclastic differentiated groups didn’t show any significant difference after 21 days. Activation of Opn and non activation of *CatK* indicated that, the DPSCs could only differentiate into osteoblasts cells, but were not able to differentiate into osteoclasts cells.

Cell viability assay with MTT showed that differentiated cells treated with osteogenic medium maintained their growth rate as comparison with control group. However, after 16 days, the growth rate decreased, but still produced viable cells. This showed that these cells still survived during differentiation into osteoblast. A decrease in growth rate may be traceable in differentiation situation; differentiated cells were weaker in their proliferation ability as compared to the control group. Statistical analysis demonstrated significant difference between the control and osteoblastic induction group after 16 days of induction (Fig 3). On the other hand, cell viability assay with MTT showed that differentiated cells treated with osteoclast medium maintained their growth rate in comparison with control group. Also, osteoclast cell produced similar growth rate as compared to control group. Our results are similar to the findings of Porter et al. (31) in which they showed that the rate of proliferation decreased, while the ALP increased in cellular bone formation and mineralization process, respectively.

## Conclusion

This study indicates that DPSC with high proliferation rate have osteoblastic differentiation capacity, but not osteoclastic differentiation. Therefore, dental pulp is a suitable for tissue repair due to the ability of these cells to generate osteoblast cells and the possibility of extracting these cells without general anesthesia.
